# Attention-Aware Patch-Based CNN for Blind 360-Degree Image Quality Assessment

**DOI:** 10.3390/s23218676

**Published:** 2023-10-24

**Authors:** Abderrezzaq Sendjasni, Mohamed-Chaker Larabi

**Affiliations:** CNRS, Université de Poitiers, XLIM, UMR 7252, 86073 Poitiers, France; abderrezzaq.sendjasni@univ-poitiers.fr

**Keywords:** image quality assessment, 360-degree images, convolutional neural networks, spatial attention, adaptive sampling, saliency-based aggregation

## Abstract

An attention-aware patch-based deep-learning model for a blind 360-degree image quality assessment (360-IQA) is introduced in this paper. It employs spatial attention mechanisms to focus on spatially significant features, in addition to short skip connections to align them. A long skip connection is adopted to allow features from the earliest layers to be used at the final level. Patches are properly sampled on the sphere to correspond to the viewports displayed to the user using head-mounted displays. The sampling incorporates the relevance of patches by considering (i) the exploration behavior and (ii) a latitude-based selection. An adaptive strategy is applied to improve the pooling of local patch qualities to global image quality. This includes an outlier score rejection step relying on the standard deviation of the obtained scores to consider the agreement, as well as a saliency to weigh them based on their visual significance. Experiments on available 360-IQA databases show that our model outperforms the state of the art in terms of accuracy and generalization ability. This is valid for general deep-learning-based models, multichannel models, and natural scene statistic-based models. Furthermore, when compared to multichannel models, the computational complexity is significantly reduced. Finally, an extensive ablation study gives insights into the efficacy of each component of the proposed model.

## 1. Introduction

The popularity of immersive environments and the large availability of dedicated devices allowed the opening of such technologies to the general public to speed up. Among the popular immersive media, 360-degree (also known as omnidirectional or spherical) images offer a captivating visual experience using either real-world scenes or computer-generated ones. Success is intimately linked to the quality of experience offered to users. Hence, appropriate measures and paradigms are of paramount importance to ensure a smooth and positive adaptation [1]. It includes quality or aesthetics of the displayed content. It also relies on the impact of technology as fatigue, comfort, immersiveness, presence, etc. This important process is referred to as the image quality assessment.

IQA is a natural and often straightforward undertaken task by human observers. This part of his acceptance process needs to be replicated for effective and challenging automation of the task. Indeed, IQA is considered as one of the most difficult image processing tasks [2], particularly perceptual quality assessment, where psychological, neurological, and computer vision aspects meet. Except for machine vision applications, the human eye is predominantly the ultimate receiver of visual signals in various real-world applications. IQA models are required to agree with the way the human visual system (HVS) processes and perceives visual signals in order to ensure reliability and accuracy. In this area, considerable efforts have been made to understand and model the perception mechanisms of the HVS for traditional content, such as 2D images. Still, many challenges arise when dealing with immersive media, including 360-degree images. Here, the nature and distinct characteristics of such content as well as the used viewing devices, i.e., HMDs, require a deeper understanding of the impact on QoE. Many factors influence 360-degree QoE assessment related to users, including immersion, presence, and cyber-sickness, and related to devices, including the field of view (FoV), resolution, and rendering [1,3]. Understanding all these factors and their influence on visual perception is paramount for developing predictive and accurate IQA methods.

As in many fields, the trend in the IQA field is the adoption of convolutional neural network (CNNs) architectures. One of the main reasons for using CNNs is related to their ability to learn discriminative and robust feature representations, ranging from low- to high-level semantic features. This characteristic potentially improves the performance of IQA models. During the past decade, several CNN models have been proposed for traditional 2D images, mainly patch-based ones [4], where the prediction is performed on individual portions of the input images rather than the entire content. Good performances were obtained on various 2D-IQA databases. The appeal of patch-based methods stems from their demonstrated effectiveness across many image-processing tasks, particularly image classification. Furthermore, by focusing on salient parts of the image, translated into patches, quality prediction tends to account for scene exploration. The main issue, however, is the lack of appropriate datasets with opinion scores per individual patch or a methodology to reliably derive them from global scores. To practically cope with this issue, current models assign the same MOS to patches derived from a single image.

In contrast to the CNN-based frameworks for 2D-IQA, 360-IQA models often rely on the multichannel paradigm [5]. It consists of several CNNs in parallel, each dealing with a region from the 360-degree image. By combining extracted features from each CNN channel, the model is trained to predict a single score per input image. This way, the unavailability of MOS per individual region is partially solved. However, such a paradigm significantly increases the complexity of the model, making it hard to train. Patch-based CNNs offer good performances while coping with the complexity of the multichannel architecture. The fact of having a single CNN helps train the model more efficiently with less computational requirement. Moreover, patch-based training implies sampling patches prior to training. The latter are used as data augmentation to make the data fed to the model richer, diverse, and sufficient, enhancing both accuracy and robustness.

This work introduces a novel patch-based CNN model for accurate blind 360-IQA. The model incorporates spatial attention, feature reuse, and a carefully designed structure to enhance its performance. The proposed framework includes an adaptive patch sampling, a patch-based training strategy, and patch-to-360-degree image quality aggregation. Extensive evaluations on three publicly available 360-IQA databases demonstrate the effectiveness of the proposed framework compared to state-of-the-art methods. The main contributions of this model can be summarized as follows:

- Introducing a novel behavior-based patch sampling strategy on the sphere, which takes inspiration from how users explore 360-degree images. This strategy addresses the issue of geometric distortion in projected content.
- Integrating spatial attention into the CNN model, allowing efficient learning of weight maps that indicate the importance of activations in feature maps. The model incorporates earlier layer features into the final stage through long skip-connections, enhancing its ability to capture relevant information.
- Proposing a unique aggregation method that combines outlier rejection and saliency to handle the variability in predicted quality scores when aggregating from patches to overall image quality. Quality scores outside an agreement range are excluded, and saliency is used to weigh selected local qualities based on their visual relevance.

## 2. Related Works

IQA models can be categorized depending on the availability of the source content as full-reference (FR), reduced-reference (NR), and no-reference (NR) [2]. Even though NR or blind methods are the most challenging ones, they are widely adopted as they reflect real-world scenarios where original images are most likely unavailable. With a focus on NR-IQA, we provide a literature review of 360-IQA. Existing NR-IQA models mainly fall into two categories: (i) natural scene statistics (NSS-based) and (ii) deep neural networks-based.

As 360-degree content gained more popularity, a few 360-IQA models have been proposed by extending traditional 2D models such as PSNR, SSIM or MSE. In particular, Yu et al. introduced the Spherical PSNR (S-PSNR) [6], which computes the PSNR on a spherical surface instead of the 2D representation. The weighted spherical PSNR (WS-PSNR) [6] uses the scaling factor of 2D-to-sphere projection as a weighting factor for PSNR estimation. Chen et al. extended the structural similarity index (SSIM) to spherical-SSIM [7] by exploiting the relationship of structural similarity between the 2D plane and the spherical domain. Hence, luminance, contrast, and structural similarities are computed at each pixel position in the spherical domain. Zakharchenko et al. proposed to compute PSNR on the Craster parabolic projection (CPP-PSNR) [8], after re-mapping pixels of both pristine and distorted images from the spherical domain to CPP. Differently, the works in [9,10] allocate weights in the computation of PSNR using saliency. These models are based on signal fidelity measurement, which does not consider characteristics of omnidirectional perception; therefore, the models cannot faithfully reflect the perceived visual quality. Moreover, they all fall in the FR category, requiring access to pristine images. Another drawback is the pixel-wise nature of these models, which does not account for the holistic aspect of the content. Consequently, the need for dedicated quality approaches for 360-IQA led to the design of a few models based on traditional NSS and structural characteristics [11,12,13,14,15,16]. Others targeted specific distortions related to 360-IQA, such as stitching [17,18], restricting their capacity to generalize to other prevalent distortions or applications.

**Figure 1:**
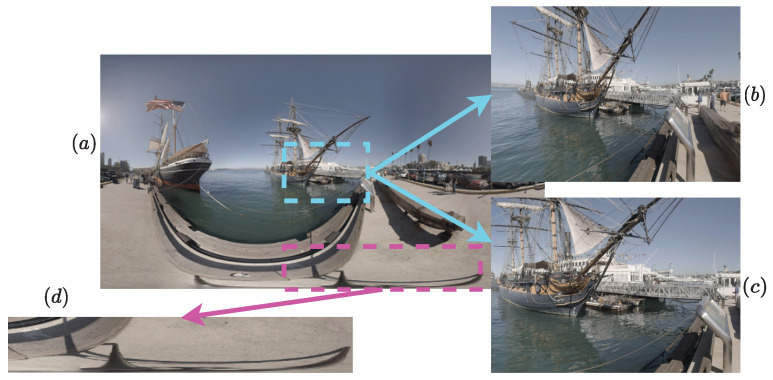
(**a**) ERP image, (**b**) radial content of the blue rectangle with a $90^{\circ }$ field of view, and (**c**) blue rectangle extracted on the ERP image. (**d**) Stretched content due to ERP.

In response to the aforementioned limitations of extended 2D models, deep-learning-based solutions have emerged [19,20,21,22,23,24,25,26,27,28,29], showing promising performances. Particularly, Sun et al. [20] introduced the multichannel paradigm by using six pre-trained ResNet-34 [30], each dedicated to one of the six faces of the cube-map projection (CMP), with a hyper architecture, where the earliest activations are combined with the last one. The six hyper ResNet-34 are used in parallel and their outputs are concatenated and regressed to a final quality score. By doing so, the lack of ground truth labels, i.e., MOS, per individual viewport, is somewhat solved as the six channels are trained to deliver a single predicted score per 360-degree image. However, it is achieved with increasing complexity due to the use of multiple CNNs. Foreseeing its performances for a 360-degree quality assessment, the multichannel paradigm has been adopted by various works. For instance, Zhou et al. [23] proposed a very similar model with shared weights among the different CNN channels. By doing so, they are identically updated according to all inputs. Here, the authors made the choice not to update the different weights of the pre-trained model Inception-V3 [31], losing the advantages of the weight sharing. Kim et al. [19] extracted 256×256 patches from the ERP image, and then fed them to parallel pre-trained models. The spherical positions of extracted patches are used along with their visual content. Thirty-two channels in total are used, each composed of a ResNet-50 and a multi-layer perceptron (MLP). The resulting model is therefore highly complex. Furthermore, predicting quality based on ERP content is inadequate since it is geometrically distorted and does not represent the content perceived by observers as illustrated in Figure 1. Hence, the polar regions are highly stretched due to sphere-to-plane projection. Moreover, the viewport content extracted on the ERP differs from that on the sphere (radial), even if both are on the same location and near the equator (the least geometrically impacted region of the image). Similarly, Truong et al. [21] used the ERP content to evaluate the quality of 360-degree images following a patch-based training scheme. Patches of 64×64 are sampled from the ERP images. During the validation, an equator-biased average pooling of patches’ scores is applied to estimate the overall quality. The same issue regarding the direct use of ERP holds for this work as well. In our previous work [24,32], we proposed to use the radial content instead of the projected one, to avoid geometric distortions related to the projection process. A multichannel network is used with extracted viewports on the sphere. The inputs are sampled according to fixations of the visual scan-path. The model learns the weight of each viewport by using visual trajectory information and just noticeable difference (JND) probability maps. Xu et al. [25] proposed the VGCN model, exploiting the dependencies among possible viewports thanks to a graph neural network (GNN) [33]. The proposed model features twenty ResNet-18 [30] as channels for the sampled viewports, in addition to a global branch based on the deep bilinear CNN (DB-CNN) [34] for ERPs. This makes the model significantly complex. Inspired by the VGCN architecture, Fu et al. [35] proposed a similar architecture, where the interaction among viewports is modeled using hyper-GNN [36]. Miaomiao et al. [26] integrated saliency prediction in the design of a CNN model combining SP-NET [37] for saliency features’ extraction, and ResNet-50 [30] for visual features’ extraction. The model is trained using CMP faces, then fine-tuned directly on ERP images. Both CMP and ERP contain distortions due to the projection process, making the adopted training strategy less consistent with the explored content. Yang et al. [22] proposed a patch-based model using the ResNet-34 [30] backbone. The input patches are enhanced using a wavelet-based enhancement CNN and then used as references to compute error maps. The selection is made randomly on ERP images, not taking into account the geometric distortion nor the relevance of the patch content. Furthermore, a simple arithmetic mean pooling is performed on the obtained patch scores to compute the final quality of the 360-degree images, overlooking the important aspect of the non-uniform distribution of perceived quality. Differently from the use of traditional CNNs, some recent works [27,29] opted for the use of ViT [38] based on the impressive results they achieved in different image processing tasks.

**Figure 2:**
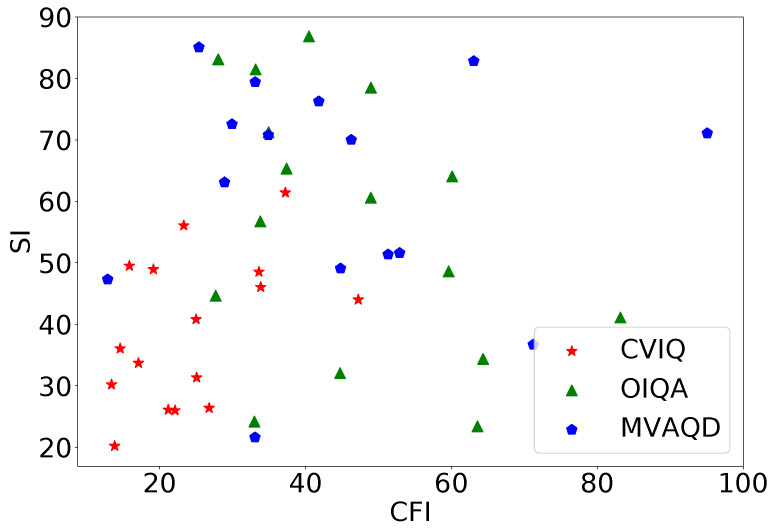
Spatial information (SI) vs. colorfulness (CFI) plot of pristine images in CVIQ, OIQA, and MVAQD databases.

As of today, 360-IQA lags behind 2D-IQA, mainly due to the lack of benchmark databases. It is of paramount importance to train, test, and validate IQA models on large and representative datasets. Unfortunately, only a few 360-IQA of them exist, and they may not be deemed sufficiently representative of the field. This is holding back the development of accurate, precise, and general-purpose 360-IQA models, especially, those based on deep learning. To the best of our knowledge, only a limited number of 360-IQA databases are proposed in the literature, including Kim et al. [19], MVAQD [11], CVIQ [20], OIQA [39], and IQA-ODI [22]. Most of these databases are not publicly available, and diversity may be lacking in some of the available ones. By analyzing the content of CVIQ, OIQA, and MVAQD in terms of spatial information (SI) and colorfulness information (CFI), one can observe a lack of diversity on CVIQ as demonstrated by the SI vs. CFI plot in Figure 2. In the meantime, SI and CFI are more spread out for OIQA and MVAQD, showing higher content diversity. SI is an indicator of edge energy and therefore used to account for spatial complexity. The CFI is a perceptual indicator of the variety and intensity of colors in images. SI and CFI are computed according to the ITU-T P.910 recommendations [40] and the metric described in [41], respectively. Such characteristics may impact the robustness (i.e., generalization) of deep learning models. To cope with these limitations, the few models proposed in the literature [19,20,23,24,26] are adopting transfer learning and fine-tuning techniques from other domains, where large and diverse data are available. The main challenge with transfer-learning is the source-to-target domain adaptation. It is acknowledged that it is more effective when the source and target domains are similar enough. The most common source domain used is image classification; hence, it is knowledge acquired by training on the ImageNet [42] dataset. The latter is composed of solely natural images with distortion occurring in the camera pipeline, which would not allow accurate predictions of perceived visual qualities for other distortions such as blur or noise. Subsequently, the design of reliable, accurate, and robust 360-IQA tools that solve the highlighted 360-IQA-related issues is in urgent need. In the following section, we describe a 360-IQA model built on the provided literature to robustly assess the quality of 360-degree images.

## 3. The Proposed Model

**Figure 3:**
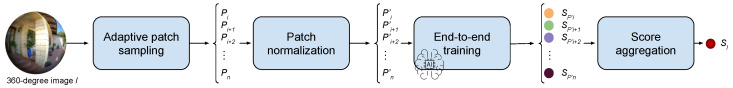
Illustration of the proposed method. Initially, the input image *I* undergoes a process of sampling into *n* individual patches, denoted as $P_{i}$. Subsequently, these patches are subjected to a normalization step, transforming them into $P^{\prime }i$. Then, predictions are generated based on these normalized patches providing quality scores $Sp_{i}^{\prime }$. Finally, a quality score $S_{I}$ is generated representing the overall quality of image *I*.

The method we propose consists of a meticulously designed series of well-defined steps, each playing a crucial role in achieving a reliable assessment, including (1) data pre-processing with patch sampling, (2) patch pixel intensities representation with normalization, (3) end-to-end training, and (4) an adaptive strategy for patch quality aggregation. A general scheme is depicted in Figure 3. In the following sections, we will delve into the intricate details of each of these steps, providing a comprehensive understanding of how our proposed method operates.

### 3.1. Input Generation

**Figure 4:**
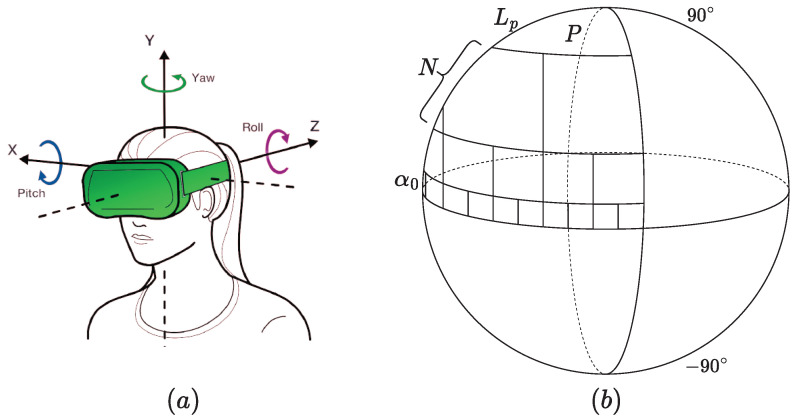
(**a**) Exploration behavior and (**b**) patch sampling based on latitude and importance of the content on the sphere.

Regions surrounding the equator of 360-degree images are known to be visually more important than polar regions. The human gaze is usually biased toward them when exploring an omnidirectional scene using HMD. Such observations were confirmed based on several psychophysical experiments [43,44]. In addition, when navigating, the user can only access the viewport in a 360-degree scene, corresponding to the currently rendered field of view (FoV) obtained from the spherical representation. The next viewport depends on head movements along the pitch (x), the yaw (y), and the roll (z), as illustrated in Figure 4a. Obviously, each viewport is perceived as a separate 2D image, where the user gaze tends to fall in the center, making it more important than the peripheral content. By taking into account this exploration behavior, it appears obvious to focus more on specific regions rather than the full scene. With this strategy, a part of the content is discarded. In contrast to naïve sampling methods used in [19,20,21,22], and to take advantage of the content diversity, we consider all the possible content by non-uniformly sampling non-overlapping patches from the sphere. The influence on the visual importance is translated by considering an adaptive sampling along the latitude (Figure 4). It is performed by considering *r* as the position of latitude and longitude equal to 0. The hemisphere sampling starts by defining $\alpha_{0}$ as the latitude of the initial patch around the equator:

(1) $$\exists \;\alpha_{0}>0\;:\;\frac{360}{\alpha_{0}}\in N^{+}\;and\;\frac{360}{\alpha_{0}.2^{N}}\in N^{+}$$

where *N* is the last level of sampling before the polar region *P*. Patches of the next level are of double size in latitude and longitude. The number of sampling levels is defined as:

(2) $$\exists \;N\geq 0\;:\left(1+\sum_{i=0}^{N}2^{i}\right)\alpha_{0}+L_{P}=90\;and\;L_{P}<\alpha_{N}$$

where $L_{P}$ is the polar region latitude.

It is widely recognized that downsampling images results in the loss of information, diminishing their significance and reducing their contribution to quality evaluation. Consequently, patches surrounding the equator are sampled with their original resolution, preserving the contained information due to their perceptual importance. As we move away from the equator, patches are sampled at twice the size of the previous patches and subsequently downsampled to match the size of the equatorial patches, denoted as $\alpha_{0}$. This approach ensures that the content is sampled with a significant level of fidelity, aligning with the exploration experienced by users wearing head-mounted displays (HMDs). Overall, $N_{patch}=2\times \left(4+\sum_{i=0}^{N}\frac{360}{2^{i}.\alpha_{0}}\right)$ non-overlapping patches are extracted from the sphere.

### 3.2. Patch Normalization

Adaptive data representation holds significant importance in machine learning solutions, particularly for CNNs. The process of image normalization prior to model training plays a pivotal role in enhancing its effectiveness. It helps the model to discern and incorporate relevant information pertinent to the specified tasks. Within the field of IQA, a commonly employed technique is divisive normalization. This approach, originally inspired by canonical computations within the neo-cortex [45] and their application in explaining neuronal responses in the primary visual cortex, has found utility as a nonlinear pre-processing step in a multitude of image processing tasks [46]. In the context of IQA, divisive normalization is primarily employed to alleviate statistical dependencies within visual signals.

In our model, we adopt a local luminance normalization approach based on divisive normalization, as recommended in [45]. It is essential to emphasize that this normalization is applied at the level of individual patches rather than being performed on the entire 360-degree image. This deliberate choice allows us to account for variations in local luminance, which can be discernible across different regions of the image. In addition, it is imperative to underscore that 360-degree images are typically constructed through the stitching of multiple captures obtained from different cameras. This process may introduce disparities in local luminance levels, underscoring the necessity of our localized approach to luminance normalization.

Our model operates by taking locally contrast-normalized patches as input. This normalization process computes the normalized value, $P^{\prime }(i,j)$, for each pixel (i,j) within a sampled patch *P* according to the following equation:

(3) $$P^{\prime }(i,j)=\frac{P(i,j)-\mu (i,j)}{\sigma (i,j)+C},$$

where μ and σ are, respectively, the mean and variance of intensity values in the normalization window. The size of this window is fixed at 3×3, a choice made to prevent any degradation in performance, as recommended in [45]. $C\in N^{+}$ is a positive constant used to avoid the calculation instability.

**Figure 5:**
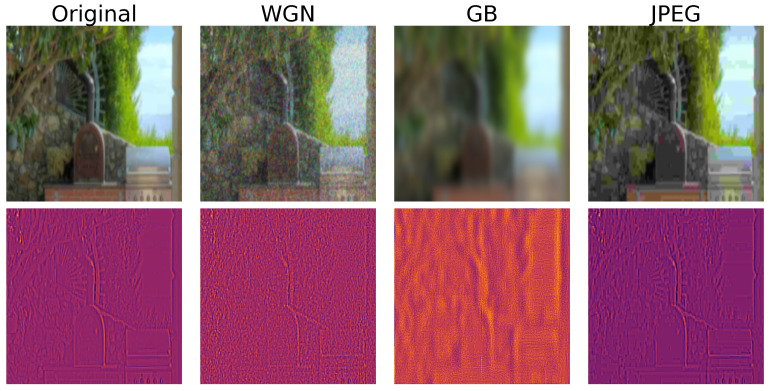
Local normalization applied on patches with different distortions.

The visual outcome of this normalization step applied to patches containing various types of distortions, as depicted in Figure 5, serves as an essential component of our methodology. Significantly, this process is adept at capturing and highlighting the presence of distortion within the analyzed patches. It particularly accentuates high-frequency details within these patches, as these details are known to wield a notable influence on the ultimate quality rating. This deliberate emphasis on high-frequency information contributes substantially to the robustness of our model, significantly enhancing its capacity to effectively assess image quality.

### 3.3. Model Architecture

**Figure 6:**
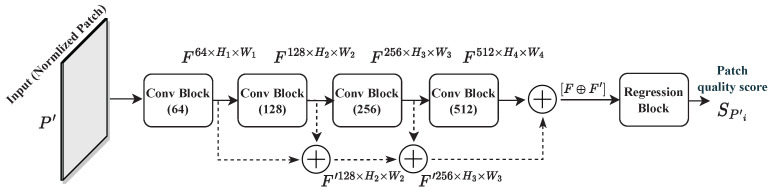
Proposed model architecture: *F* and $F^{\prime }$ are feature maps, and $S_{P_{i}^{{}^{\prime }}}$ represents the predicted quality score of patch $P^{\prime }$.

**Figure 7:**
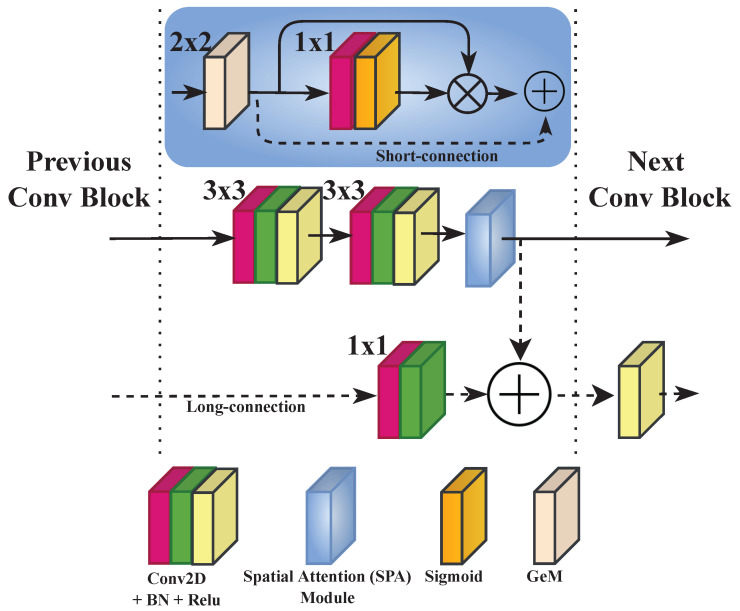
Architecture of the Conv Block with ⊗ element-wise multiplication and ⊕ element-wise addition. The 3×3 and 1×1 correspond to the kernel sizes for the convolution layers, and 2×2 represents the stride for the pooling layer (GeM).

The architecture of the proposed model is illustrated in Figure 6. In contrast to state-of-the-art quality models for 360-degree images, the proposed model adopts patch-wise training. Therefore, an architecture with a single deep CNN is designed rather than a multichannel one. The latter is found to be highly complex, requiring more computational resources, and is difficult to train. The proposed model is composed of four convolutional blocks (Conv Block) with a doubling number of filters, ranging from 64 to 512. This way, the CNN model can learn more discriminative features and be able to achieve a better representation of these features [4]. Each block is composed of two Conv layers with 3×3 filter size, each followed by a batch normalization (BN) layer [47], and then a rectified linear activation function (ReLU) [48]. The structure of the Conv Block is depicted in Figure 7. BN is used to make the model faster, stable, and more robust to bad initialization [47]. It is recommended to place BN right after the Conv layers and before the activation function, which helps to produce activations with a stable distribution [47]. All convolutions are used with zero-padding to preserve more features and produce an output of the same dimension as the input.

Right after the second activation (ReLU) in each Conv Block, a spatial attention (SPA) module is used. The latter outputs a refined feature map $F\in R^{D_{n}\times H_{n}\times W_{n}}$, where *D*, *H*, and *W* stand for the height, width, and dimension in terms of the number of channels of the deep features F, respectively, and *n* corresponds to the number of Conv Blocks. Here, the *“spatial”* term refers to the domain space embedded within each feature map. Therefore, spatial attention represents the attention mechanism, as known as the attention mask, on the learned feature maps. It conveys what is important to learn and to focus on within each feature map. The composition of the SPA module is detailed in Figure 7 (blue rectangle). It includes a three-fold sequential operation. The first consists of a pooling stage in order to encode and capture highly discriminative features before applying the attention. Commonly, max- or average-pooling is applied [22] at this stage, or together in some cases as in [49]. Differently, the proposed SPA module applies the generalized mean pooling (GeM) [50], which generalizes the pooling equation for each feature map $X_{D}$ of $F\in R^{D\times H\times W}$ as follows:

(4) $$y={\left(\frac{1}{|X_{D}^{s}|}\sum_{x\in X^{s}}x^{p_{D}}\right)}^{\frac{1}{p_{D}}},$$

where *y* represents the aggregated value, $X_{D}$ the set of values, *s* the pooling stride of 2×2, and $p_{D}\in \left[1,\infty \right)$ is the hyperparameter that controls the pooling for each feature map $X_{D}$. When $p_{D}=1$, the GeM corresponds to the average pooling. When $p_{D}\to \infty$, the GeM corresponds to the max pooling. As the GeM pooling is a differentiable operation as stated in [50], $p_{D}$ can be considered as a trainable parameter. This helps update it through back-propagation. Hence, the SPA module exploits the states between the average and max pooling by relying on back-propagation to learn the hyperparameter $p_{D}$, allowing it to use the adequate pooling method. The second part consists of creating the spatial attention mask $M_{SPA}\left(F\right)\in R^{H\times W}$ by means of the sigmoid activation function σ(.), as follows:

(5) $$M_{SPA}\left(F\right)=\sigma \left(Conv^{1\times 1}\left(\left[GeM^{2\times 2}\left(F\right)\right]\right)\right)$$

$M_{SPA}\left(F\right)\in R^{H\times W}$ is generated using the aggregated maps obtained by GeM(.). First, $F\in R^{D\times H\times W}$ goes through a Conv layer with 1×1 kernel size and a single filter, resulting in $F\in R^{1\times H\times W}$. The idea is to reduce the dimensionality of the generated feature maps by creating a single-channel one. Then, the sigmoid activation function σ(.) is applied to the output of the Conv layer. As the sigmoid is a probabilistic activation function, it will map all the values of the input $F\in R^{1\times H\times W}$ to a range between 0 and 1, generating the spatial attention mask $M_{SPA}\in R^{H\times W}$. The latter is then applied to all the feature maps in the output of GeM(.) using the element-wise product as denoted in Equation (6).

(6) $$F_{SPA}\in R^{D\times H\times W}=M_{SPA}⊗GeM^{2\times 2}\left(F\right)$$

**Figure 8:**
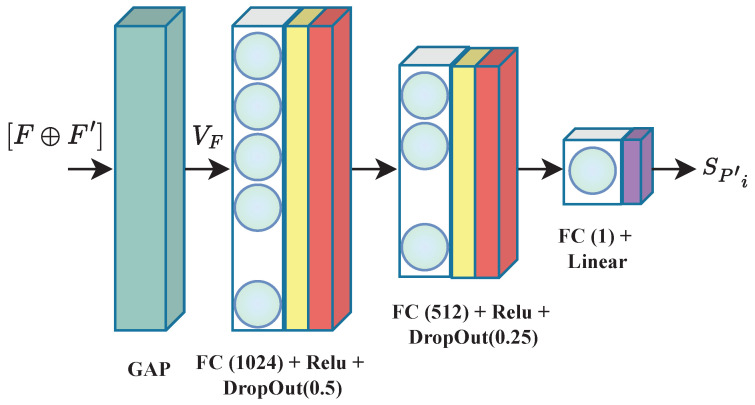
Architecture of the used regression block. “GAP” corresponds to global average pooling, and “FC” stands for a fully connected layer. $V_{F}$ is the generated feature vector.

Within each SPA module, a short skip-connection is implemented, connecting the output of GeM(.) with the refined feature map $F_{SPA}\in R^{D\times H\times W}$. Here, the connection is accomplished by means of element-wise addition, as illustrated in Figure 7. As a result, the aligned features will be greater compared to non-aligned ones. The SPA output is obtained as:

(7) $$F\in R^{D\times H\times W}=ReLU\left(F_{SPA}⊕GeM^{2\times 2}\left(F\right)\right),$$

is first fed to the next Conv Block in a feed-forward fashion. Additionally, it is acknowledged that the earliest convolutions in a CNN capture low-level features, whereas latter convolutions focus on high-level semantic features. In addition, the HVS is highly sensitive to low-level features, such as spatial frequency, line orientation, texture, and contrast [51]. The use of such features at later stages in the CNN could have the potential to improve the model’s performances and stability for various image processing tasks [20,52]. Therefore, the proposed model implements a long skip-connection by means of hierarchical element-wise additions at each Conv Block. Here, $F\in R^{D\times H\times W}$ are hierarchically added together into $F^{\prime }\in R^{D\times H\times W}$, as follows:

(8) $$F_{i}^{\prime }=ReLU\left(F_{i}^{\prime }⊕Conv_{filter=filter_{i}}^{1\times 1}\left(F_{i-1}\right)\right),$$

where *i* denotes the Conv Block number. The $Conv^{1\times 1}$ with the same filter number as the block *i* is required to match the dimension for the addition operation. At the last Conv Block, both F and $F^{\prime }$ are added together. Finally, global average pooling (GAP) is used to reduce the spatial dimensions of the encoded feature maps by generating a feature vector $V_{F}\in R^{D\times 1\times 1}$. This operation is known to decrease over-fitting, and is accomplished as follows:

(9) $$y^{c}=\frac{1}{N}\sum_{i,j}{\left[F⊕F^{\prime }\right]}_{i,j}^{c},$$

where $y^{c}$ is the output value of feature map ${\left[F⊕F^{\prime }\right]}^{c}$ at channel *c*, and (i,j) is the pixel index of the corresponding feature value. The feature vector $V_{F}$ obtained by the GAP operation is fed to the quality regression block, where the obtained features are combined to estimate the quality score. The regression block is illustrated in Figure 8 and is composed of two FC layers with dimensions of 1024 and 512, respectively, each followed by a ReLU activation function and a dropout layer for regularization. A final FC layer with a single node and a linear activation is added to deliver the final quality score. Weight initialization in the model is performed according to He et al. [53] to start the training with a Gaussian probability distribution. The latter helps the model to avoid numerical difficulties due to unstable initial weights [54].

### 3.4. Loss Function

Generally, IQA is considered a regression task. The commonly used loss functions are the mean square error (MSE) and mean absolute error (MAE). However, it is known that MAE is less sensitive to outliers in the data, but it is not differentiable at zero, whereas, MSE is differentiable everywhere, but it is highly sensitive to outliers. The Huber loss [55] combines the best characteristics of both loss functions. It is both differentiable everywhere and robust to outliers. Therefore, the proposed model uses the Huber loss, defined as follows:

(10) $$L_{\delta }\left(y,S_{P^{\prime }{}_{i}}\right)=\left\{\begin{array}{cc} \frac{1}{2}{\left(y-S_{P^{\prime }{}_{i}}\right)}^{2} & \mathrm{for}\;\left|y-S_{P^{\prime }{}_{i}}\right|\leq \delta ,\\ \delta \left|y-S_{P^{\prime }{}_{i}}\right|-\frac{1}{2}\delta^{2} & \mathrm{otherwise}, \end{array}\right.$$

where *y* is the ground truth score (MOS) and $S_{P^{\prime }{}_{i}}$ is the predicted one. $\delta \in R^{+}$ controls the use of either MAE or MSE. Its value is defined as 1.35 based on [55].

### 3.5. Quality Scores’ Aggregation

As a patch-based strategy, the proposed model predicts a set of quality scores LQs corresponding to *N* sampled patches from each 360-degree image *I*. To derive the quality of the whole 360-degree image, one may apply a simple and straightforward average of LQs. However, it is widely admitted that the quality of a scene is non-uniformly distributed, meaning that certain regions are more contributing to global quality than others. This is even more true for 360-degree images. Moreover, the perceived quality is highly affected by the most distorted regions among the selected ones [56], as the human gaze tends to fall on these salient regions when exploring a scene. A simple arithmetic mean cannot express such aspects, notwithstanding the non-uniformly distribution of quality. Giving the same importance to all local qualities by averaging LQs may not be consistent with (i) the scene exploration and (ii) the quality distribution.

**Figure 9:**
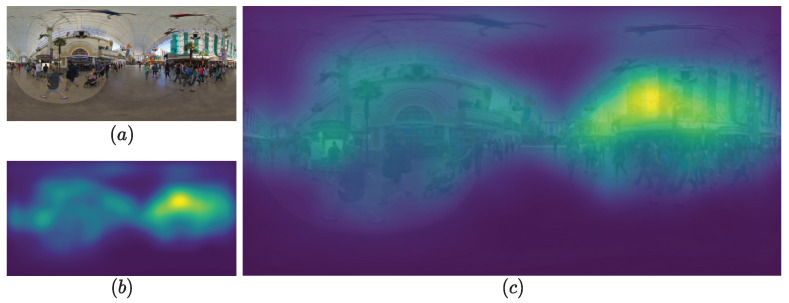
Illustration of saliency map to highlight important regions. (**a**) A 360-degree image, (**b**) its saliency, and (**c**) their superposition.

In response to the limitation of simply averaging LQs, the proposed model uses visual saliency to weigh local qualities in LQs. In this regard, it considers the relevance of salient regions over less salient ones by means of visual attention. Hence, for each 360-degree image *I*, a saliency map $Sal_{I}$ is generated using the 360-degree saliency model proposed by Xia et al. [57]. Figure 9 illustrates the obtained saliency map of a 360-degree image. As is shown, important regions are highlighted and mostly located around the equator, as explained earlier. We have chosen this model due to its straightforward nature and its broad availability in the field of predicting saliency in 360-degree images. Alternative models could also be considered, since this step serves as a post-processing step.

By taking saliency into consideration, patches in salient regions contribute significantly to the global quality score with high weight values. $S_{I}$ is then computed by weighting the predicted local qualities in LQs for patches sampled from the image *I* using the estimated weights $W_{i}$ with i∈{1,2,…,N}, and *N* is the number of patches.

(11) $$S_{I}=\frac{\sum_{i=1}^{N}W_{i}\times S_{P_{i}^{\prime }}}{\sum_{i=1}^{N}W_{i}},$$

where the weights are obtained as follows:

(12) $$W_{i}=\frac{\sum_{R_{P}}Sal_{P_{i}}{\left(k\right)}^{\;\mathrm{if}\;Sal_{P_{i}}\left(k\right)\geq th}}{\sum_{R_{I}}Sal_{I}\left(k\right)},$$

$Sal_{P_{i}}$ represents the saliency region corresponding to the extracted patch $P_{i}$, and $R_{P}$ and $R_{I}$ represent the resolution of *P* and *I*, respectively. Differently from standard approaches where the weights are the summation of pixel intensities within a patch [58], we compute the ratio with respect to the overall saliency of the image *I*. By doing so, the weights will reflect the importance of the local quality of *P* with regard to the global quality of *I*. Moreover, only values greater than a threshold th are summed together, considering only higher saliency values. th is defined empirically as the 75^th^ percentile of intensity values in $Sal_{P_{i}}$.

Prior to weighting local qualities by visual saliency, we implement the principle of outliers’ rejection (OR) from LQs. Scores falling far from the median of LQs are discarded in order to only aggregate those within an agreement interval. This solution is motivated by subjective ratings, in which only scores that concur are considered to compute the MOS. Outliers are detected using the standard deviation σ(.) of LQs. In this case, a score is identified as an outlier only if it falls outside the interval [−λ∗σ(LQs),+λ∗σ(LQs)], where $\lambda \in R^{+}$ is a parameter used to determine the appropriate agreement range with respect to the variability among LQs. Therefore, probable prediction errors are corrected by fine-tuning the λ parameter, accounting for statistical properties of each LQs. To the best of our knowledge, this is the first work incorporating such properties to aggregate quality scores for IQA in general. Formally, the final score $S_{I}$ is obtained as:

(13) $$S_{I}=\frac{\sum_{i\in |s|}W_{i}\times S_{p_{i}}}{\sum_{i\in |s|}W_{i}},\;\;\;\mathrm{with}\;\;\;s=\pm \lambda *\sigma \left(LQs\right).$$

## 4. Experiments and Results

### 4.1. Experimental Setup

#### 4.1.1. Datasets

Training and evaluation of the proposed model are performed on three publicly available 360-IQA datasets, namely OIQA [39], CVIQ [20], and MVAQD [11]. We conducted the performance comparison with state-of-the-art models on the OIQA and CVIQ databases. Performances of SOTA models are gathered from the corresponding papers or other studies, due to the unavailability of most training codes. As MVAQD is rarely used in the literature, we discarded it from the comparison, but it was included in the ablation study. Details regarding these databases are summarized in Table 1.

#### 4.1.2. Implementation Details

The proposed model is implemented using TensorFlow and trained on a server equipped with Intel Xeon Silver 4208 2.1 GHz, 192 G RAM, and a GPU Nvidia Tesla V100S 32 G. The batch size was set to 32 and the Adam optimizer [59] is used with $\beta_{1}=0.9$ and $\beta_{2}=0.999$, following the recommendation in [59] so as to prevent adverse effects on optimization. The learning rate is set initially to 1e−3 with a learning decay of MM1e−4/EPOCHS to help the optimization of the model. A five-fold cross-validation is adopted for a complete assessment on the selected datasets. Each fold was trained for 100 epochs. During training, the datasets are randomly separated into 80% for training (including 20% dedicated for validation) and 20% for testing. To ensure a complete separation of the training and testing sets, the distorted images linked to the same pristine source are allocated to the same set. This helps to test the model on unseen content, avoiding a very common mistake that is frequently overlooked, in which datasets are split based on distorted images. Therefore, the model’s assessment is made on already-observed content, resulting in unreliable assessments.

#### 4.1.3. Evaluation Metrics

Three commonly used performance evaluation criteria recommended by the ITU [60] are used to evaluate the performances of our model, including the Pearson linear correlation coefficient (PLCC) to evaluate the accuracy, Spearman rank-order correlation coefficient (SRCC) for monotonicity, and the root–mean-squared error (RMSE) to evaluate the prediction errors. A PLCC or SRCC values close to 1 indicate good performance, whereas values close to 0 indicate poor performance. In terms of RMSE, lower values correspond to less prediction errors.

In addition to PLCC, SRCC, and RMSE, we use an additional metric proposed by Krasula et al. [61]. This metric provides more insight on the behavior and performances of an IQA model. It examines the statistical significance among several models by comparing the area under the curve (AUC) values from the receiver operating characteristic (ROC) analysis, mostly known for binary classification tasks. Hence, the ability to classify images according to their perceived quality can be highlighted. Basically, it compares the capacity to distinguish different/similar and better/worse among image pairs and provides a percentage of correct classification denoted as $C_{0}$. Unfortunately, such a metric necessitates additional data, such as the standard deviation of MOS. For this study, we could only collect the required data for the OIQA dataset. Thus, we only perform this analysis on that specific dataset.

As one of the main motivations behind the development of the proposed model is to achieve better accuracy while having less complexity compared to state-of-the-art models in general and multichannel ones in particular, we performed an inference analysis. Hence, a complexity analysis is provided in terms of the model’s parameters and the number of floating-point operations (FLOPs). The latter provides insights on the computations required by the model. A large number of FLOPs implies a higher complexity, suggesting a longer calculation time. Since the inference analysis is independent of the training, we used a different hardware configuration. A computer equipped with an Intel^®^ Cor^TM^ i9-9880H @ 2.30 GHz, 32 GB of RAM, and an Nvidia Quadro T2000 MAX-Q 4 GB GPU is used to measure the computational complexity.

### 4.2. Performance Comparison with SOTA Models

With the aim to illustrate the effectiveness of the proposed model, a comparison with sixteen state-of-the-art IQA models, including 2D- and 360-IQA models, is performed. For each category, traditional and deep-learning (DL)-based models are selected. The selected models include PSNR, SSIM, MS-SSIM [62], FSIM [63], BRISQUE [64], and BMPRI [65], DB-CNN [34], and DipIQ [66], representing 2D-IQA models. S-PSNR [6], WS-PSNR [6], SSP-BOIQA [11], Yun et al. [12], MC360IQA [20], Zhou et al. [23], VGCN [25], AHGCN [35], and S^3^DAVS [16], representing 360-IQA models. MC360IQA, Zhou et al., VGCN, and AHGCN are all deep learning-based solutions using the multichannel paradigm with a varying number of channels, from six to twenty, making them highly complex models.

The overall and per individual distortion performances in terms of PLCC, SRCC, and RMSE are summarized in Table 2 for OIQA and Table 3 for CVIQ. The performances of the proposed model are reported as the median of the five folds.

From the performances obtained on OIQA (Table 2), one can observe that the proposed model achieves the best overall performance compared to both 360- and 2D-IQA models. This observation is valid regardless of the used aggregation of the local qualities method, demonstrating its superiority over SOTA models. In particular, multichannel-based models, where the proposed model outperformed the MC360IQA in terms of PLCC (respecting SRCC) by approximately 5% (specifically, 4.7%), Zhou et al. by approximately 8% (specifically, 4.2%), VGCN by approximately 1.4% (specifically, 1.2%), and AHGCN by approximately 0.7% (specifically, 0.4%). Similar behavior can be observed with the prediction error in terms of RMSE. This illustrates the accuracy and monotonicity of our model and its ability to evaluate the quality of 360-degree images in a close manner to human judgment. Regarding the performances across different types of distortions, the SOTA models still lag behind the performances of the proposed model for JPEG and GB. For JP2K and WGN, the proposed model achieves competitive performances compared to VGCN, AHGCN, and Yun et al. in terms of PLCC and SRCC. A possible explanation could be the LCN normalization. In terms of RMSE, however, the proposed model achieved the lowest error for individual distortions as for the overall database. The superiority of the proposed model is highlighted once more. When looking at the performance of 2D-IQA models, it is clear that DB-CNN outperforms 2D traditional models, including FR models such as SSIM and MS-SSIM. This demonstrates the advantages of a deep learning-based model for quality assessment over traditional approaches.

Among the performances of the proposed model, the best is achieved when using outlier rejection (OR) and saliency together as the aggregation strategy. It appears that discarding predicted local qualities that are outside the agreement range ±λ∗σ(LQs) before averaging the scores improves the correlation performances. This is demonstrated by the acquired performances on the overall database and across distortions compared to considering all local qualities, except for GB. Simple averaging resulted in slightly better correlation performances for GB. Such behavior can be explained by the fact that the model agrees more with the quality of blurred patches compared to other distortions. When using saliency to weight-predicted local qualities according to the importance of their corresponding patches after discarding outliers, a similar behavior is observed. It is known that saliency is affected by distortions in general and blur in particular, since the content is smoothed. Hence, using saliency as a weighting strategy is not contributing significantly to the GB distortion. It is to be noted that the discrepancy between the three variants of the model is within a small range of 0.15%.

Table 3 further summarizes the performance results of the proposed model as well as SOTA models overall and on each distortion type of CVIQ. The first observation that emerges is that overall, the best performances are obtained by deep learning-based 360-IQA models. Considering all content of CVIQ, the proposed model achieved slightly worse in terms of accuracy compared to VGCN while outperforming MC360IQA, Zhou et al., and AHGCN. In terms of monotonicity, our model achieved the best correlation. As for the prediction errors, the MC360IQA scored the best, with a slight difference. With regard to the performances obtained on OIQA (Table 2), those on CVIQ seem to be slightly worse when compared to SOTA models. Here, the diversity of the training dataset is affecting the accuracy of the model. It is worth noting that CVIQ is less diverse compared to OIQA in terms of (i) distortion types and (ii) content, which is paramount to better training the model. Regarding the performances across the different distortion types, the proposed model reached competitive performances compared to multichannel ones. Despite less diversity on CVIQ, the proposed model still performs well. For JPEG distortion, some 2D models appear to perform well, such as SSIM and FSIM. The latter obtained the best accuracy with images compressed using H.265/HEVC. Such an achievement could be explained by the fact that these models have access to pristine images.

The proposed model’s correlation performance appears to be influenced by the aggregation strategy. Except for H.265/HEVC distortion, the average solution resulted in the best outcomes, making adaptive pooling less effective on CVIQ. This is closely tied to the nature of the content. As the OR-based aggregation decreased the performances, it implies that the predicted local qualities are mostly close to the median value. Therefore, less diversity exists among LQs.

**Figure 10:**
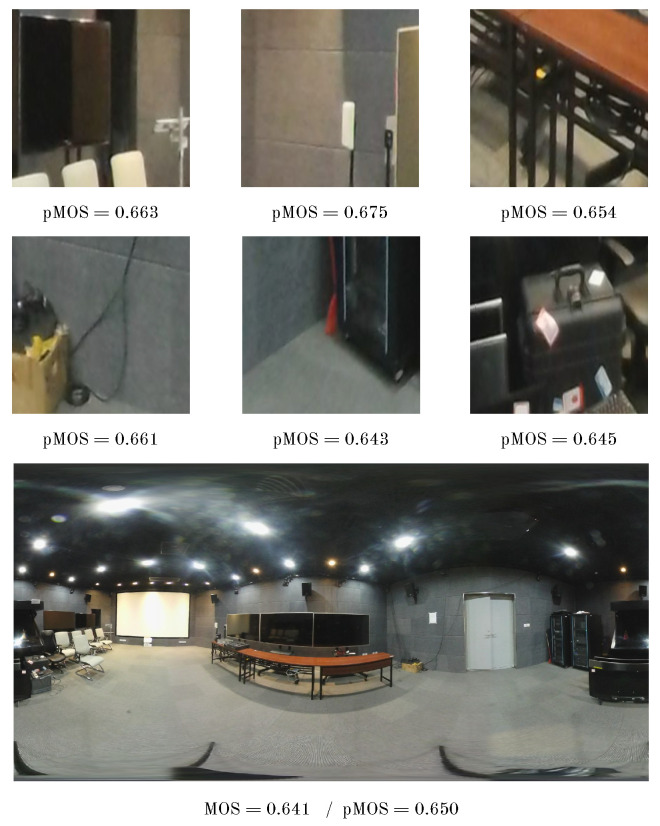
Illustration of prediction using the proposed method from patches to 360-degree image.

In Figure 10, we present a visual representation of the prediction process as employed in our proposed method. This process entails a transition from the examination of individual patches to the comprehensive evaluation of an entire 360-degree image. As illustrated, each patch is assigned a distinct quality score, demonstrating relatively low variability among these scores. The aggregation of these individual patch scores leads to a closely aligned predicted MOS, which closely mirrors the actual MOS.

### 4.3. Cross-Dataset Evaluation

With the intent to demonstrate the generalization ability of the proposed model to new content built using different conditions, we carried out a cross-dataset evaluation. Hence, we trained our model on OIQA and tested its performance on CVIQ and vice versa. Table 4 reports the obtained performances in terms of accuracy (PLCC) and monotonicity (SRCC). As highlighted, our model outperforms the others while showing the lowest computational complexity, demonstrating its ability to generalize new content and distortions. For instance, by training on OIQA and testing on CVIQ, our model achieved a PLCC (respecting SRCC) score of 0.9145 (specifically, 0.9020), outperforming MC360IQA by approximately 10% PLCC and 7% SRCC, VGCN by approximately 3% PLCC and 4% SRCC, and Zhou et al. by approximately 8% PLCC and 9% SRCC. By training on CVIQ and testing on OIQA, similar behavior can be observed. Comparing the training datasets, we observe that training on OIQA resulted in significantly better performances. Here, the diversity in terms of content, as well as distortion types, appears to significantly contribute to the generalization capability. Whereas, with less diversity as in the case of CVIQ, relatively poor performances are attained. Moreover, OIQA comprises GB and WGN, which are not available on CVIQ. This affects the ability of the models to adapt to new distortions. Nevertheless, the proposed model attained satisfactory performances compared to SOTA.

In terms of complexity, the proposed model with 6.19 million trainable parameters and 3.38 G of FLOPs has drastically fewer parameters and requires the least operations compared to the other models. In particular, VGCN (26.7 M, 220 G) requires much more computational complexity. This is due to its architecture, involving twenty ResNet-18 in parallel with a graph CNN and a sub-network composed of the DB-CNN. In the case of MC360IQA (22.4 M, 22.7 G) and Zhou et al. (29.3 M, 6.45 G), a higher complexity is also shown. However, for the latter, it is significantly lower in terms of the number of FLOPs compared to the other multichannel models. The reason lies in the weight sharing among the CNN channels.

**Figure 11:**
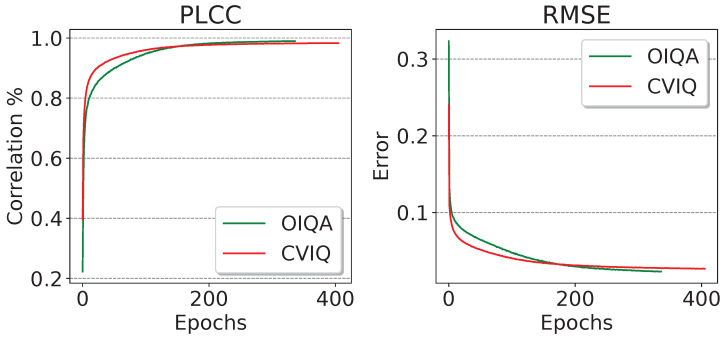
Evolution of PLCC and RMSE during the training of the proposed model on OIQA and CVIQ.

We further monitored the evolution of PLCC and RMSE performances of the proposed model during training on the whole OIQA and CVIQ databases to analyze the behavior. From Figure 11, we first observe that training on OIQA (336 epochs) converges faster than CVIQ (407 epochs). This is due to the size of the training sets composed of 528 images for CVIQ and 320 for OIQA (40% difference). Moreover, PLCC and RMSE seem to improve at the same time in both databases, until reaching a tie by the 150th epoch for PLCC and 170th for RMSE. Overall, the results obtained on both databases are fairly competitive, with OIQA marginally outperforming. This actively exhibits the proposed model’s ability to represent the training data as well as to generalize new ones.

### 4.4. Ablation Study

We conduct an ablation study to analyze the impact of various components composing our model, including (i) patch sampling, (ii) the use of the SPA module, and short and long skip-connections, (iii) loss functions by comparing the use of the Huber loss to MSE and MAE, and (iv) quality score aggregation. As mentioned previously, we use the MVAQD database in addition to OIQA and CVIQ.

#### 4.4.1. Sampling Methods

**Figure 12:**
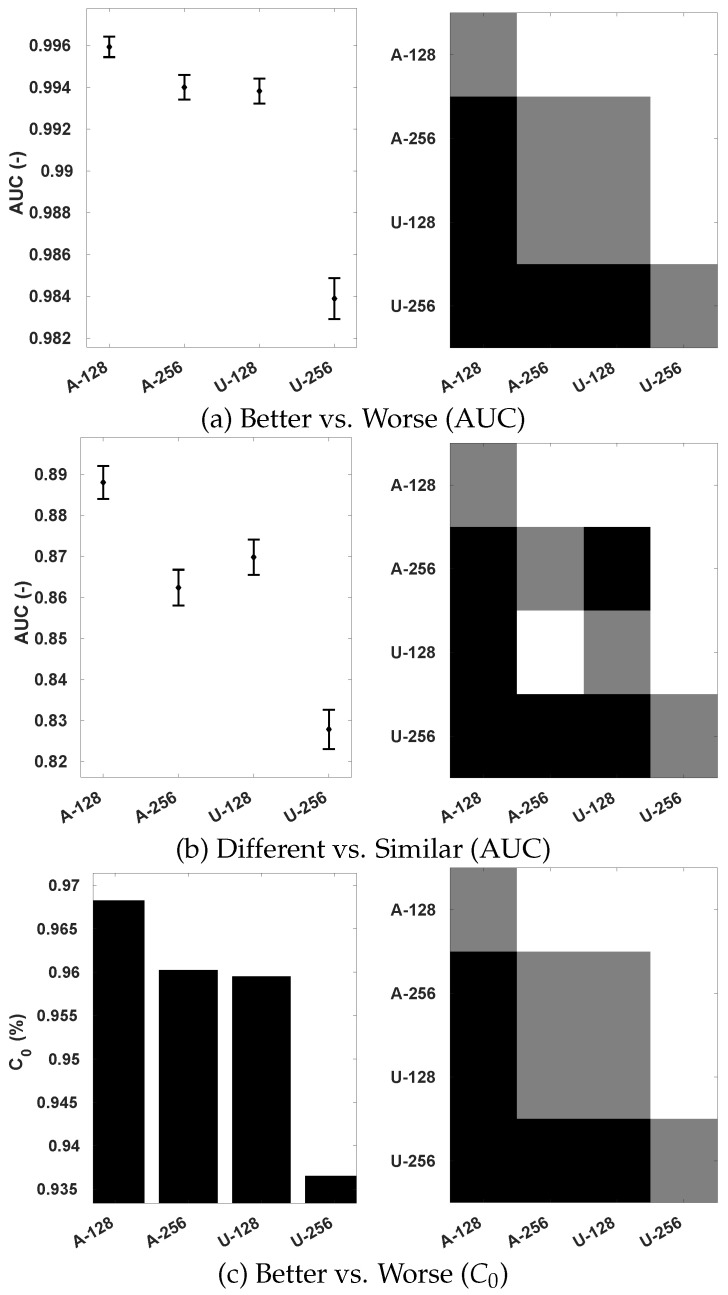
Statistical significance analysis of sampling methods on OIQA depending on $\alpha_{0}$ using Krasula et al. method. “A-128/A-256” stands for adaptive sampling with $\alpha_{0}=128/256$ and “U-128/U-256” for uniform sampling. For the significance plots, white/black square: row model is statistically better/worse than column one; gray square: statistically indistinguishable.

With the intent to evaluate the efficacy of using an adaptive sampling strategy and demonstrate the advantage of considering 360-IQA specific characteristics, we compare its performance with a uniform sampling strategy. The latter uniformly samples patches from the sphere without considering their importance. Here, all patches are sampled with the same size $\alpha_{0}$ (described in Section 3.1) along the latitude and longitude. In addition, we further analyze the influence of patch size on the performance of the model. We set $\alpha_{0}$ to 128 and 256 pixels. The correlations performances are provided in Table 5, and the statistical significance analysis conducted on OIQA (because of the availability of the standard deviation of MOS) is illustrated in Figure 12.

From Table 5, the first emerging observation is that the overall performance of uniform sampling slightly lags behind that of adaptive sampling. Incorporating 360-IQA inherent properties to sample patches on the sphere appears to improve the performance of the model. The adaptive sampling improves the predictions monotonicity of our model, translated by almost 10% of an increase in terms of SRCC when $\alpha_{0}=256$ on MVAQD. Regarding the influence of the size $\alpha_{0}$, the setting $\alpha_{0}=128$ outperformed $\alpha_{0}=256$ despite the used sampling method, except on CVIQ. Using $\alpha_{0}=256$ resulted in a slight improvement in terms of SRCC and RMSE. By setting $\alpha_{0}=128$, more patches can be obtained, creating a richer training set with more examples compared to setting $\alpha_{0}=256$. The amount of training data certainly matters because it affects the accuracy of the model. Looking closely into the case of CVIQ with $\alpha_{0}=256$, one might assume that fewer patches introduce less redundancy as all patches from the same image are labeled with the same MOS. This is also depicted by the small margin in performances on OIQA that are within 1% of improvement. However, by analyzing the statistical significance on OIQA in terms of the ability to distinguish and classify the stimulus with better quality among image pairs, setting $\alpha_{0}=128$ is found to be significantly superior, as illustrated with the plots in Figure 12. From these plots, several observations can be made. First, the scores obtained by both Better vs. Worse (Figure 12a,c) analysis are significantly higher compared to the different vs. similar (Figure 12b) one. A difference up to approximately 10% can be observed with the error bars, which represent the 95% confidence intervals. This indicates that distinguishing between different/similar pairs remains a difficult challenge when compared to better/worse pairs, corresponding to the visual quality judgment made by the HVS. Second, the use of adaptive sampling with $\alpha_{0}=128$ significantly outperforms the other three settings, proving the superiority of (i) incorporating 360-IQA characteristics and (ii) generating larger training sets. Based on the overall assessment and statistical significance analysis, we chose $\alpha_{0}=128$ with the adaptive sampling for training the proposed model.

#### 4.4.2. SPA Module and Skip-Connections

Each convolutional block in the proposed model is augmented with a spatial attention (SPA) module, as outlined in Section 3.3. The latter is required to assist the model in focusing on significant features. This is mostly accomplished by the learnable weights via back-propagation, which optimizes the attention masks $M_{SPA}\left(F\right)\in R^{H\times W}$ at each step. The results of the ablation study are provided in Table 6 to evaluate the efficacy of the SPA module and the utilization of short- and long-skip connections. In addition, a computational complexity analysis is performed, and the results are provided in the same table. The computational time for prediction is obtained as the average times across 100 images. The comparison is conducted with the SAL-360IQA [67], a previous version of the proposed model, where min-, max-, and average-pooling are employed at each Conv Block instead of the SPA module.

The performances in Table 6 suggest that the proposed model is robust. An accuracy up to 0.97, 0.96, and 0.95 expressed by the PLCC is obtained on OIQA, CVIQ, and MVAQD, respectively. The remaining evaluation metrics exhibit similar behavior, supporting its robustness. This demonstrates the effectiveness of the adopted training strategy, including adaptive patch sampling, normalization, and model design. When comparing the proposed model and its version without the SPA module and skip-connections, the latter reaches the best performance overall, while exhibiting less complexity. The complexity is decreased by approximately 50%, 42%, and 39% in terms of #Params, #FLOPs, and prediction time, respectively, as illustrated by the complexity analysis.

**Figure 13:**
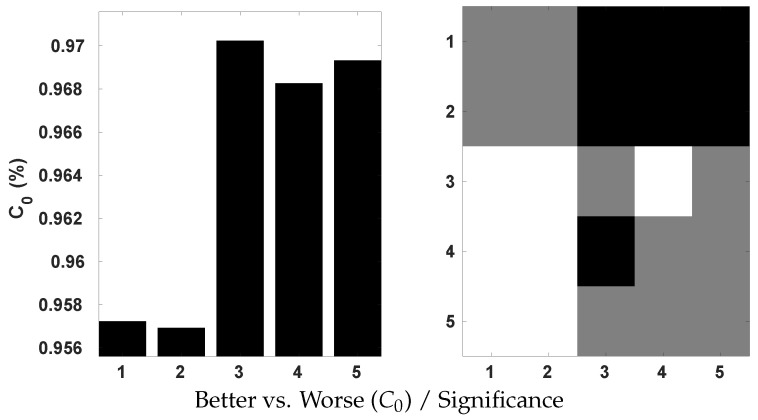
Statistical significance analysis on OIQA for the SPA module/skip-connections [61]. 1: No SPA/No skip, 2: SPA/No skip, 3: SPA/Short, 4: SPA/Long, and 5: SPA/Short + Long.

The ablation study on the use of skip-connections revealed a significant improvement of the performances as depicted by Figure 13, especially the short-connection of the SPA module. Aligning each feature map (before applying attention) with its refined version (after the attention), makes the aligned weights greater compared to the non-aligned ones. This helps to better highlight the important features in the spatial dimension of each feature map. In the case of long-connections, it appears that a significant improvement is reached. Here, reusing the earliest features at the last stage brought additional information that could be lost between the first Conv Block and the last one. When compared to short-connections, the latter is adding more values to the overall performance of the model. Using only short- or long-connection, the model is able to classify image pairs into better/worse significantly better than when using the long-connection only. However, combining both skip-connections yielded no statistically significant difference when compared to using only short-connections. Moreover, when combining short and long skip-connections with the SPA module, the model achieves the best overall performances, as highlighted in Table 6. As a result, the latter is adopted as our model’s final architecture.

**Figure 14:**
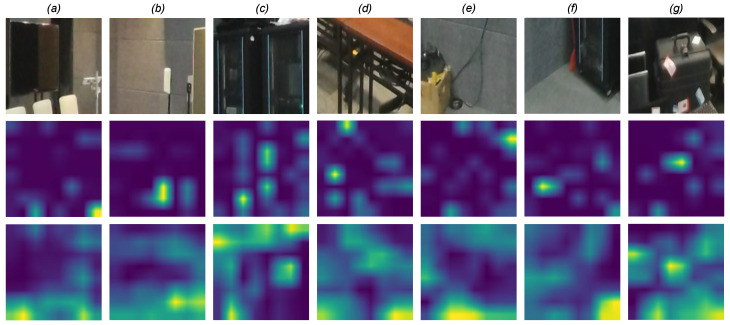
Visualizations of Grad-CAM activation maps with/without SPA module (respecting **second/third row**) applied on patches (**first row**).

In addition to the quantitative results, we provide a visualization of the activation maps using the Grad-CAM technique [68] in Figure 14. This visualization helps identify the influential regions in estimating the quality at the patch level. By analyzing the Grad-CAM heatmaps generated for a set of patches from the same image, we can clearly observe the highlighted regions that contribute the most. Analyzing the heatmap, one can notice that only a portion of the patches is considered, indicating a local selection of visual features for the model’s decision. Comparing the Grad-CAM visualization with and without the use of the SPA module, we can observe that the salient regions are highly refined and concentrated when incorporating the SPA module. Without the SPA module, the activation maps appear more spread-out. For example, in the second row of the visualization, patches (*b*) and (*g*) show a high refinement of the salient regions, specifically focusing on the white button and tags on the suitcase. This refined attention to salient details reflects human perception. However, for patch (*e*), the SPA module failed to focus on the vacuum, which is a less prominent object in the patch. It is important to note that Grad-CAM provides local explanations, offering insights into the model’s decision at the pixel and patch levels.

#### 4.4.3. Loss Functions

**Figure 15:**
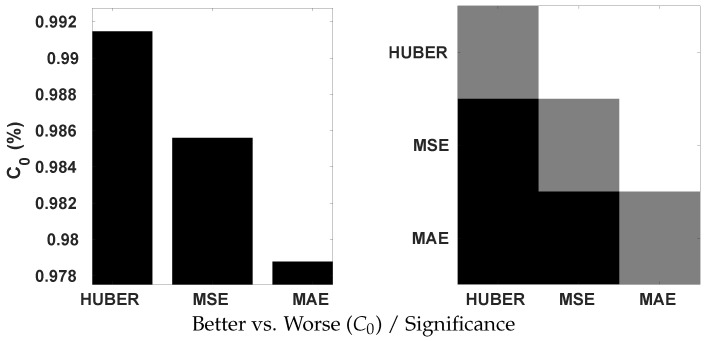
Statistical significance analysis on OIQA of Huber, MSE, and MAE loss functions using Krasula et al. method.

The Huber loss, which combines the properties of MSE and MAE, was applied to train our model. To ascertain its effectiveness, we conduct a comparative analysis with the use of MSE and MAE by the Krasula et al. method on OIQA. The results are illustrated on Figure 15 in terms of the percentage of correct classifications into better/worse pairs, denoted as $C_{0}$. According to the provided plots, the Huber loss appears to be significantly superior to MSE and MAE based on both AUC different/similar and $C_{0}$ better/worse analysis. This actively demonstrates its interest in the contribution to the accuracy of the model. It is proven that better/worse classification is less challenging than different/similar classification. Yet, an accuracy of approx. 0.93 is achieved with the Huber loss compared to 0.91/0.90 with MSE/MAE. Moreover, MSE performed significantly better than MAE for the AUC different/similar while being statistically indistinguishable for $C_{0}$ better/worse.

**Figure 16:**
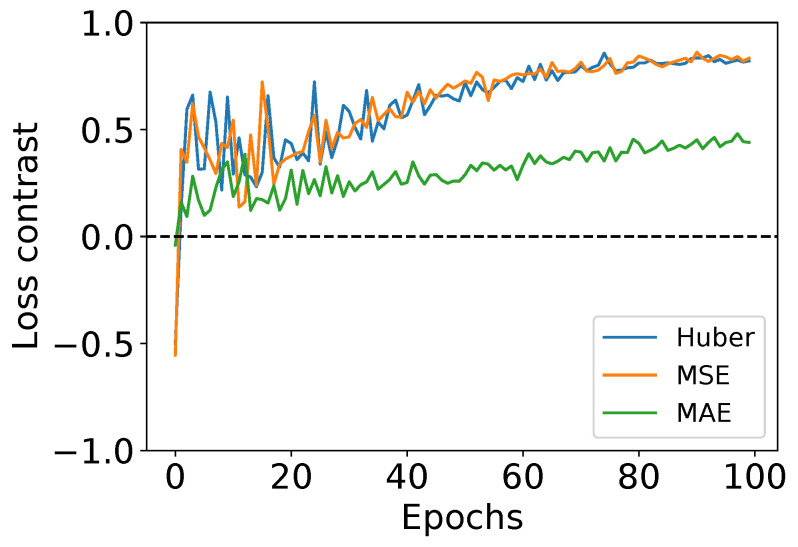
Contrast (val_loss−loss)/(val_loss+loss) between training and validation losses, (0 → equal loss).

In addition to the performance comparison, in Figure 16 we provide the contrast between the training and validation losses to analyze their evolution. The contrast is computed as (val_loss−loss)/(val_loss+loss). The latter provides insights on the training behavior, including (1) under-fitting happening when it can neither model the training data nor generalize the new one, (2) over-fitting when a model learns the details and noise in the training data to the extent that it negatively impacts its performance on new data, and (3) good-fit when reaching stable learning. A contrast equal to 0 depicts an equal loss between training and validation, whereas a contrast equal or close to 1 suggests an important gap between both losses, with val_loss being higher and the opposite if equal or close to −1. From the curves, no sign of under-fitting can be noticed regardless of the used loss function, depicting the efficacy of the adopted training strategy. The MAE appears to have less gap between the training/validation losses, compared to Huber or MSE. The behavior of the latter can be explained by the fact that the training loss is improving faster than the validation one. However, the achieved losses by the Huber loss are smaller than MSE and MAE, with an important margin compared to MAE. For instance, the Huber loss attained a training/validation losses of 0.0003/0.003 compared to MSE:0.0005/0.006 and MAE:0.024/0.071 for the same fold. This supports the observations drawn above from the statistical significance analysis.

#### 4.4.4. Local Qualities’ Aggregation

A patch-based CNN model is basically trained on *N* individual patches extracted from the input images. This means that the model is trained only on these patches, without any cue about the 360-degree content. Therefore, *N* local qualities corresponding to *N* patches are predicted. The mapping of these individual scores to a single quality score is somewhat challenging. This operation must be performed by the adaptive aggregation to improve the correlation with the human judgment of perceived quality. As described in Section 3.5, the proposed model uses an aggregation strategy based on OR.

**Figure 17:**
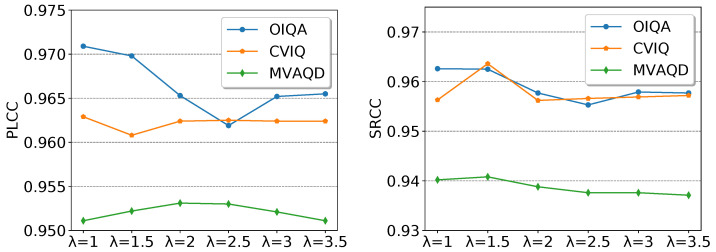
Evaluating the performances of the OR-based local qualities’ aggregation by varying the value of λ.

To investigate the effect of the λ parameter on the models’ performances, we conducted a comparative analysis in terms of PLCC and SRCC on OIQA, CVIQ, and MVAQD. The performances are shown in Figure 17. PLCC is strongly affected by the value of λ. This is particularly true on OIQA, where PLCC shows a relative inflection after λ=2.5. The same can be observed with SRCC. As for CVIQ, PLCC appears to be stable and less affected by λ. This supports the observation drawn in Section 4.2, where the predicted local qualities seemed to be within an agreement. However, SRCC expresses an increase at λ=1.5, whereas it is stable elsewhere. For MVAQD, both PLCC and SRCC slightly improve at different λ values before decreasing. The obtained results highlighted an important aspect related to the dataset dependency.

## 5. Conclusions

In this paper, an attention-aware patch-based CNN model for blind 360-IQA was presented. Spatial attention is used to help the model focus on spatially meaningful features. Skip-connections within the spatial attention module were also integrated to align the preserved features using spatial attention. The exploration behavior, as well as a latitude-based selection, were used to sample patches appropriately on the sphere for training the model. The latter has shown a significant improvement over standard sampling, not accounting for 360-IQA peculiarities. Patch quality aggregation was accomplished adaptively using saliency and outlier rejection, which resulted in an improved correlation with the ground truth. The proposed model demonstrated good performances across different databases and distortions. In comparison to SOTA models in general, and multichannel ones in particular, our model reached competitive or much better performances while offering the lowest complexity. This demonstrates the value of the adopted (i) appropriate patch sampling, (ii) data representation, (iii) model architecture, and (iv) aggregation strategy. Furthermore, the proposed model’s generalization capacity demonstrated its superiority in adapting to new content and distortion-based cross-database evaluations.

While the proposed strategy led to some improvements, several questions are required to be addressed, especially the adaptive patch labeling when using a patch-based model. The lack of mean opinion scores per individual patch is an important problem to tackle in our future work.

## Figures and Tables

**Table 1:** Summary of the used 360-degree image databases.

Database	OIQA [39]	CVIQ [20]	MVAQD [11]
Ref images	16	16	15
Distorted images	320	528	300
Distortion type (Distortion levels)	JPG (5)/WGN (5) JP2K (5)/BLR (5)	JPG (11)/AVC (11) HEVC (11)	BLR (4)/HEVC (4) JPG (4)/JP2K (4) WGN (4)
# of subjects	20 (M: 15, F: 5)	20 (M: 14, F: 6)	26 (M:16, F: 10)
HMD	HTC Vive	HTC Vive	HTC Vive Pro

**Table 2:** Performance comparison with SOTA models on OIQA. The best performance is highlighted in **bold** and the second-best is underlined. Ours_Avg_, Our_OR_, and Our_OR+SAL_ stand for the proposed model with average, OR-based, and OR + saliency-based aggregation of local qualities, respectively.

			Overall			JPEG			JP2K			WGN			GB		
	Ref.	Model	PLCC↑	SRCC↑	RMSE↓	PLCC↑	SRCC↑	RMSE↓	PLCC↑	SRCC↑	RMSE↓	PLCC↑	SRCC↑	RMSE↓	PLCC↑	SRCC↑	RMSE↓
2D-IQA	FR	PSNR	0.6910	0.6802	10.388	0.8658	0.8291	7.8570	0.8492	0.8421	7.9357	0.9317	0.9008	4.6392	0.6357	0.6374	10.250
	FR	SSIM	0.8892	0.8798	6.5814	0.9409	0.9346	5.3193	0.9336	0.9357	5.3829	0.9026	0.8846	5.4965	0.9188	0.9238	5.2404
	FR	MS-SSIM	0.8427	0.8332	7.7442	0.9312	0.9188	5.7214	0.9265	0.9267	5.6560	0.9672	0.9484	3.2460	0.8623	0.8624	6.7250
	FR	FSIM	0.9274	0.9225	8.2501	0.9478	0.9351	7.3215	0.9545	0.9573	6.5924	0.9466	0.9176	6.0670	0.9444	0.9478	6.4372
	NR	BRISQUE	0.8424	0.8331	11.261	0.9160	0.9392	8.9920	0.7397	0.6750	15.082	0.9553	0.9372	3.4270	0.8663	0.8508	9.6970
	NR	BMPRI	0.6503	0.6238	15.874	0.9160	0.8954	7.8861	0.8322	0.8214	12.280	0.9611	0.9490	3.5340	0.5199	0.3807	12.248
	NR	DB-CNN	0.8852	0.8653	9.7172	0.9755	**0.9607**	4.9350	0.9770	**0.9786**	3.8324	0.9772	**0.9786**	3.8323	0.9536	0.8865	5.8752
	NR	DipIQ	0.7012	0.6917	10.259	0.8291	0.7891	8.7833	0.9165	0.9182	6.0300	0.9556	0.9432	3.7742	0.9321	0.8983	4.8161
360-IQA	FR	S-PSNR	0.7153	0.7115	10.052	0.8703	0.8285	7.7319	0.8555	0.8489	7.7811	0.9190	0.8846	5.0329	0.6929	0.6917	9.5736
	FR	WS-PSNR	0.6985	0.6932	10.294	0.8607	0.8278	7.9919	0.8435	0.8322	8.0719	0.9221	0.8853	4.9415	0.6609	0.6583	9.9652
	NR	SSP-BOIQA	0.8600	0.8650	7.3131	0.8772	0.8345	7.6201	0.8532	0.8522	7.5013	0.9054	0.8434	5.4510	0.8544	0.8623	6.8342
	NR	Yun et al.	0.9437	0.9369	7.1911	0.9612	0.9536	6.3330	0.9697	0.9676	5.3941	0.9789	0.9737	3.8453	0.9645	0.9558	5.1582
	NR	S^3^DAVS	0.9405	0.9348	7.1830	0.9267	0.8872	8.6340	0.9306	0.7717	9.6230	0.9725	0.9623	4.3840	0.9692	0.9662	4.8050
	NR	MC360IQA	0.9247	0.9187	4.6247	0.9279	0.9190	4.5058	0.9324	0.9252	4.5825	0.9344	0.9345	3.7908	0.9220	0.9353	4.5256
	NR	Zhou et al.	0.8991	0.9232	6.3963	0.9363	0.9405	5.6911	0.9200	0.9343	5.8862	0.9682	0.9570	3.3304	0.9252	0.9200	4.9721
	NR	VGCN	0.9584	0.9515	5.9670	0.9540	0.9294	6.7201	0.9771	0.9464	4.7721	**0.9811**	0.9750	3.4932	0.9852	0.9651	3.3270
	NR	AHGCN	0.9649	0.9590	5.4871	0.9649	0.9276	5.8860	**0.9820**	0.9643	4.2360	0.9706	**0.9786**	4.3410	0.9756	0.9759	4.2640
	NR	Ours_Avg_	0.9668	0.9585	3.5707	0.9741	0.9429	3.5956	0.9731	0.9607	**3.4249**	0.9755	0.9643	2.8710	**0.9880**	**0.9786**	**2.1828**
		Ours_OR_	0.9709	0.9626	3.3538	0.9763	0.9571	3.4221	0.9737	0.9536	3.3843	0.9761	0.9607	2.8373	0.9865	**0.9786**	2.2843
		Ours_OR+SAL_	**0.9721**	**0.9629**	**3.3317**	**0.9779**	0.9500	**3.3125**	0.9741	0.9536	3.3587	0.9764	0.9714	**2.7005**	0.9866	**0.9786**	2.2388

**Table 3:** Performance comparison with SOTA models on the CVIQ database. The best performance is highlighted in **bold** and the second-best is underlined. Ours_Avg_, Our_OR_, and Our_OR+SAL_ stand for the proposed model with average, OR-based, and OR + saliency-based aggregation of local qualities, respectively.

			Overall			JPEG			H.264/AVC			H.265/HEVC		
	Ref.	Model	PLCC↑	SRCC↑	RMSE↓	PLCC↑	SRCC↑	RMSE↓	PLCC↑	SRCC↑	RMSE↓	PLCC↑	SRCC↑	RMSE↓
2D-IQA	FR	PSNR	0.7320	0.7662	9.0397	0.7342	0.8643	8.5866	0.7572	0.7592	8.0448	0.7169	0.7215	8.3279
	FR	SSIM	0.8857	0.8972	6.2140	0.9334	0.9749	3.7986	0.9451	0.9457	4.0165	0.9220	0.9232	4.6219
	FR	MS-SSIM	0.8762	0.8875	6.4836	0.9140	0.9628	4.6101	0.8794	0.8805	5.8583	0.8604	0.8610	6.1165
	FR	FSIM	0.934	0.9152	4.8964	0.9839	0.6939	2.8928	0.9534	0.9439	4.0327	**0.9617**	0.9532	3.9239
	NR	BRISQUE	0.7448	0.7641	9.0751	0.8489	0.9091	7.1137	0.7193	0.7294	8.4558	0.7151	0.7104	8.4646
	NR	BMPRI	0.7919	0.7470	8.5258	0.9874	0.9562	2.5597	0.7161	0.6731	9.3318	0.6154	0.6715	9.3071
	NR	DB-CNN	0.9356	0.9308	4.9311	0.9775	0.9576	3.3862	0.9564	0.9545	3.9063	8.6460	0.8693	5.3350
	NR	DipIQ	0.7060	0.6232	9.9601	0.9285	0.7930	6.3530	0.6203	0.6353	9.6954	0.3611	0.6366	11.216
360-IQA	FR	S-PSNR	0.7467	0.7741	8.9066	0.7520	0.8772	8.1974	0.7690	0.7748	7.8743	0.7389	0.7428	8.0515
	FR	WS-PSNR	0.7498	0.7755	8.8816	0.7604	0.8802	8.1019	0.7726	0.7748	7.8143	0.7430	0.7469	7.9974
	NR	SSP-BOIQA	0.8900	0.8561	6.9414	0.9155	0.8533	6.8471	0.8850	0.8611	7.0422	0.8544	0.8410	6.3020
	NR	Yun et al.	0.9481	0.9344	4.4964	0.9844	0.9650	2.9910	0.9576	0.9545	3.5623	0.9289	0.9278	4.4534
	NR	S^3^DAVS	0.9533	0.9426	4.1022	0.9707	0.9302	3.8675	0.9586	0.9447	3.3925	0.9367	0.8802	4.5675
	NR	MC360IQA	0.9506	0.9139	**3.0935**	0.9746	0.9316	2.6388	0.9461	0.9244	**2.6983**	0.9126	0.8985	**3.2935**
	NR	Zhou et al.	0.9020	0.9112	6.1170	0.9572	0.9611	5.6014	0.9533	0.9495	3.8730	0.9291	0.9141	4.5252
	NR	VGCN	**0.9651**	0.9639	3.6573	**0.9894**	**0.9759**	**2.3590**	0.9719	0.9659	3.1490	0.9401	0.9432	4.0257
	NR	AHGCN	0.9643	0.9623	3.6990	0.9869	0.9686	2.6162	**0.9793**	**0.9753**	2.7084	0.9419	0.9412	3.9657
	NR	Ours_Avg_	0.9645	**0.9642**	3.3398	0.9835	0.9475	3.0334	0.9736	0.9716	2.8318	0.9419	0.9535	3.9149
		Ours_OR_	0.9629	0.9563	3.8672	0.9835	0.9535	3.0337	0.9719	0.9733	2.9205	0.9389	0.9525	3.9872
		Ours_OR+SAL_	0.9630	0.9611	3.8789	0.9843	0.9578	3.1435	0.9735	0.9686	2.8048	0.9502	**0.9566**	**3.6326**

**Table 4:** Cross-database performances and complexity comparison of the proposed model with SOTA models (best performance is highlighted in **bold**).

		Trained/Tested on		Complexity	
		OIQA/CVIQ	CVIQ/OIQA	# Params	# FLOPs
MC360IQA	PLCC↑	0.8249	0.7443	22.4 M	22.7 G
	SRCC↑	0.8442	0.6981		
VGCN	PLCC↑	0.8886	0.7911	26.7 M	220 G
	SRCC↑	0.8629	0.7832		
Zhou et al.	PLCC↑	0.8470	0.7350	29.3 M	6.45 G
	SRCC↑	0.8250	0.7410		
Ours_OR+SAL_	PLCC↑	**0.9145**	**0.8029**	**6.19 M**	**3.38 G**
	SRCC↑	**0.9020**	**0.7926**		

**Table 5:** Ablation of sampling methods wi patch size ($\alpha_{0}$).

	Sampling	Uniform		Adaptive	
	$\alpha_{0}$	128 px	256 px	128 px	256 px
OIQA	PLCC↑	0.9600	0.9572	**0.9612**	0.9607
	SRCC↑	0.9524	0.9491	**0.9563**	0.9553
	RMSE↓	**3.8535**	4.2070	3.9344	3.9890
CVIQ	PLCC↑	0.9592	0.9587	**0.9618**	0.9603
	SRCC↑	0.9390	0.9401	0.9553	**0.9570**
	RMSE↓	3.7900	3.8417	3.9227	**3.7665**
MVAQD	PLCC↑	0.9450	0.8940	**0.9512**	0.9409
	SRCC↑	0.9022	0.8371	**0.9402**	0.9253
	RMSE↓	**0.2809**	0.9399	0.3296	0.3899

**Table 6:** Ablation of the SPA module and skip-connections. Best performance in **bold** and the second-best is underlined.

		OIQA			CVIQ			MVAQD			Computational Complexity		
Version	Skip-Conx	PLCC↑	SRCC↑	RMSE↓	PLCC↑	SRCC↑	RMSE↓	PLCC↑	SRCC↑	RMSE↓	#Params↓	#FLOPs↓	Time↓
SAL-360IQA	✗	0.9611	0.9547	3.8950	0.9589	0.9531	3.8308	0.9507	0.9428	0.3311	9.89 M	5.15 G	0.52s
Ours	✗	0.9618	0.9537	3.9118	**0.9627**	0.9555	**3.6519**	0.9428	0.9296	0.3839	**5.74 M**	**3.34 G**	0.38s
	Short	0.9664	0.9583	3.6214	0.9615	0.9534	3.7132	**0.9546**	**0.9468**	0.3430	**5.74 M**	**3.34 G**	**0.35s**
	Long	0.9647	0.9567	3.6818	0.9565	0.9587	3.9390	0.9474	0.8863	0.3437	6.19 M	3.38 G	0.36s
	Short+Long	**0.9668**	**0.9585**	**3.5707**	0.9607	**0.9634**	3.7475	0.9512	0.9402	**0.3296**	6.19 M	3.38 G	0.36s

## Data Availability

Not applicable.
